# World No Tobacco Day — May 31, 2014

**Published:** 2014-05-30

The global tobacco epidemic contributed to 100 million deaths worldwide during the 20th century and continues to kill nearly 6 million persons each year, including approximately 600,000 from secondhand smoke. If current trends persist, an estimated 500 million persons alive today will die from the use of tobacco products. By 2030, tobacco use will result in approximately 8 million deaths worldwide each year. About 80% of these preventable deaths will occur in low- and middle-income countries (1,2).

Sponsored by the World Health Organization and observed worldwide on May 31 each year, World No Tobacco Day highlights the health risks of tobacco use and promotes effective actions to reduce tobacco consumption. This year, World No Tobacco Day calls on countries to raise taxes on tobacco (2).

Increasing the price of tobacco products by raising tobacco taxes is one of the most powerful and cost-effective means to prevent and reduce tobacco use, but it is an underused strategy (3,4). Research shows that higher taxes can reduce the relative affordability of tobacco products, encourage smokers to quit, reduce cigarette consumption, and discourage young persons from smoking initiation. It also generates government revenues, which can be invested in effective tobacco control efforts that will further reduce tobacco use (3,4).
